# Redetermination of (+)-methamphetamine hydro­chloride at 90 K

**DOI:** 10.1107/S1600536808011550

**Published:** 2008-04-30

**Authors:** Patrick Hakey, Wayne Ouellette, Jon Zubieta, Timothy Korter

**Affiliations:** a Department of Chemistry, Syracuse University, Syracuse, New York 13244, USA

## Abstract

The title crystal structure (systematic name: *N*-methyl-1-phenyl­propan-2-aminium chloride), C_10_H_16_N^+^·Cl^−^, was orginally determined by Simon, Bocskei & Torok [*Acta Pharm. Hung.* (1992). **62**, 225–230] and Yao, Kan & Wang [*Huaxue Shijie* (1999). **40**, 568–570] at room temperature but no atomic coordinates are available for these determinations. The mol­ecule has inter­est with respect to biological activity. In the crystal structure, inter­molecular N—H⋯Cl hydrogen bonds form one-dimensional chains.

## Related literature

For related literature, see: Cho (1990 ▶); Cho & Melega (2002 ▶); Davis & Swalwell (1994 ▶); O’Neil *et al.* (2001 ▶); Simon *et al.* (1992 ▶); Yao *et al.* (1999 ▶); Yu *et al.* (2003 ▶).
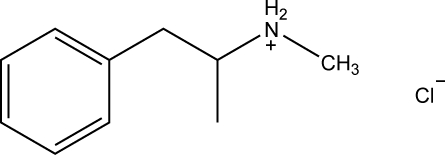


## Experimental

### 

#### Crystal data

C_10_H_16_N^+^·Cl^−^
*M* *_r_* = 185.69
Monoclinic, 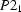

*a* = 7.1022 (11) Å
*b* = 7.2949 (11) Å
*c* = 10.8121 (17) Å
β = 97.293 (4)°
*V* = 555.64 (15) Å^3^
*Z* = 2
Mo *K*α radiation
μ = 0.30 mm^−1^
*T* = 90 (2) K
0.28 × 0.14 × 0.10 mm

#### Data collection

Bruker APEX CCD area-detector diffractometer
Absorption correction: multi-scan (*SADABS*; Bruker, 2002 ▶) *T*_min_ = 0.922, *T*_max_ = 0.971
5892 measured reflections
2720 independent reflections
2379 reflections with *I* > 2σ(*I*)
*R*_int_ = 0.047

#### Refinement

*R*[*F* ^2^ > 2σ(*F* ^2^)] = 0.051
*wR*(*F* ^2^) = 0.117
*S* = 1.05
2720 reflections
174 parameters
1 restraint
All H-atom parameters refined
Δρ_max_ = 0.43 e Å^−3^
Δρ_min_ = −0.46 e Å^−3^
Absolute structure: Flack (1983 ▶), 1235 Freidel pairs
Flack parameter: 0.00 (10)

### 

Data collection: *SMART* (Bruker, 2002 ▶); cell refinement: *SAINT* (Bruker, 2002 ▶); data reduction: *SAINT*; program(s) used to solve structure: *SHELXS97* (Sheldrick, 2008 ▶); program(s) used to refine structure: *SHELXL97* (Sheldrick, 2008 ▶); molecular graphics: *CrystalMaker* (Palmer, 2006 ▶); software used to prepare material for publication: *SHELXTL* (Sheldrick, 2008 ▶).

## Supplementary Material

Crystal structure: contains datablocks I, global. DOI: 10.1107/S1600536808011550/lh2608sup1.cif

Structure factors: contains datablocks I. DOI: 10.1107/S1600536808011550/lh2608Isup2.hkl

Additional supplementary materials:  crystallographic information; 3D view; checkCIF report

## Figures and Tables

**Table 1:** Hydrogen-bond geometry (Å, °)

*D*—H⋯*A*	*D*—H	H⋯*A*	*D*⋯*A*	*D*—H⋯*A*
N1—H1*D*⋯Cl1^i^	0.93 (4)	2.14 (4)	3.069 (2)	179 (4)
N1—H1*E*⋯Cl1^ii^	0.90 (3)	2.22 (3)	3.116 (2)	176 (3)

Symmetry codes: (i) 
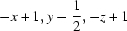
; (ii) 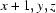.

**Fig. 1.**
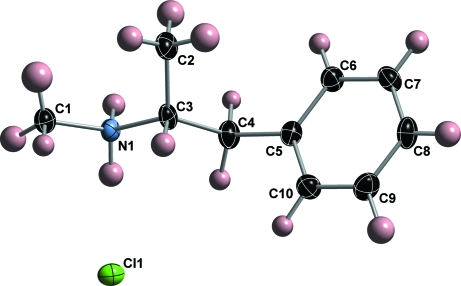
The molecular structure of the title compound, with the atom numbering scheme and thermal ellipsoids drawn at 50% probability level.

**Fig. 2.**
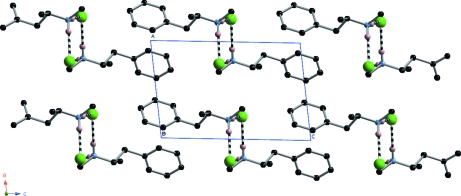
The crystal packing of the title compound viewed in the ac plane, showing hydrogen bonds as dashed lines. H atoms not involved in hydrogen bonding have been omitted.

**Fig. 3.**
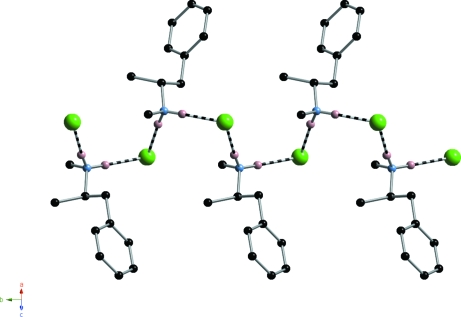
The crystal packing of the title compound, showing hydrogen bonds as dashed lines. H atoms not involved in hydrogen bonding have been omitted.

## supplementary crystallographic information

### Comment

The compound, (+)-methamphetamine hydrochloride has been reinvestigated in this study by single-crystal *x*-ray diffraction to provide a complete determination of the atomic coordinates and lattice dimensions at 90 (2) K. Earlier structural studies on this compound by Simon *et al.* (1992) and by Yanhong *et al.* (1999) were performed at or near room temperature and did not include atomic coordinates. The determination of crystallographic data at cryogenic temperatures improves the precision of the atomic coordinates and also provides insight into temperature-induced lattice changes. This information is important in the complete understanding of the molecular solid and is particularly useful for validation of first-principles solid-state modeling.

The compound studied is a synthetic sympathomimetic drug and is specified as a controlled substance by the United States Federal government (O'Neil *et al.*, 2001). The substance is a strong stimulant that affects the central nervous system (*CNS*) and contributes cardiactoxicity (Yu *et al.*, 2003). The use of methamphetamine has increased substantially and is becoming a problem nation wide with its use increasing across all age groups (Cho & Melega, 2002). The compound has a more potent effect on the *CNS* than structurally similar amphetamine due to its increased penetration of the *CNS* (Davis & Swalwell,1994). The potency of methamphetamine is also dependent upon its chirality, as its dextrorotatory enantiomer exhibits an effect roughly fives times greater than that provided by the levorotatory enantiomer (Cho, 1990). The stimulant effects of methamphetamine can be compared to the effects brought on by the use of cocaine, however, the duration of the effects can be much greater for the methamphetamine than for cocaine (Cho, 1990).

The (+)-methamphetamine hydrochloride form of methamphetamine has become the primary form used (Cho & Melega, 2002). This highlights the importance of complete characterization of the substance. Knowledge of the solid-state *Crystal Structure* of this compound is imperative for its identification and detection *via* various spectroscopic methods, such as solid-state NMR and terahertz. The unit-cell dimensions determined by this study are slightly smaller than those published by Simon *et al.*, (1992) leading to a reduction in the unit cell volume of approximately 2.4% from the previously calculated value. Overall the basic structural parameters, such as the space group, *P*2_1_, are in agreement with earlier work (Simon *et al.*, 1992).

### Experimental

The material for this work was purchased from Sigma-Aldrich and was used without any further purification.

### Refinement

H atoms were located in a difference map and refined freely.

### Crystal data

**Table:**

C_10_H_16_N^+^·Cl^–^	*F*_000_ = 200
*M**_r_* = 185.69	*D*_x_ = 1.110 Mg m^−^^3^
Monoclinic, *P*2_1_	Mo *K*α radiation λ = 0.71073 Å
Hall symbol: P 2yb	Cell parameters from 964 reflections
*a* = 7.1022 (11) Å	θ = 2.9–22.5º
*b* = 7.2949 (11) Å	µ = 0.30 mm^−^^1^
*c* = 10.8121 (17) Å	*T* = 90 (2) K
β = 97.293 (4)º	Block, colorless
*V* = 555.64 (15) Å^3^	0.28 × 0.14 × 0.10 mm
*Z* = 2	

### Data collection

**Table:**

Bruker APEX CCD area-detector diffractometer	2720 independent reflections
Monochromator: graphite	2379 reflections with *I* > 2σ(*I*)
Detector resolution: 512 pixels mm^-1^	*R*_int_ = 0.047
*T* = 90(2) K	θ_max_ = 28.2º
φ and ω scans	θ_min_ = 1.9º
Absorption correction: multi-scan(SADABS; Bruker, 2002)	*h* = −9→9
*T*_min_ = 0.922, *T*_max_ = 0.971	*k* = −9→9
5892 measured reflections	*l* = −14→14

### Refinement

**Table:**

Refinement on *F*^2^	Hydrogen site location: inferred from neighbouring sites
Least-squares matrix: full	All H-atom parameters refined
*R*[*F*^2^ > 2σ(*F*^2^)] = 0.051	*w* = 1/[σ^2^(*F*_o_^2^) + (0.0575*P*)^2^] where *P* = (*F*_o_^2^ + 2*F*_c_^2^)/3
*wR*(*F*^2^) = 0.117	(Δ/σ)_max_ < 0.001
*S* = 1.05	Δρ_max_ = 0.43 e Å^−^^3^
2720 reflections	Δρ_min_ = −0.46 e Å^−^^3^
174 parameters	Extinction correction: none
1 restraint	Absolute structure: Flack (1983), 1235 Freidel pairs
Primary atom site location: structure-invariant direct methods	Flack parameter: 0.00 (10)
Secondary atom site location: difference Fourier map	

### Special details

**Table:**

Geometry. All e.s.d.'s (except the e.s.d. in the dihedral angle between two l.s. planes) are estimated using the full covariance matrix. The cell e.s.d.'s are taken into account individually in the estimation of e.s.d.'s in distances, angles and torsion angles; correlations between e.s.d.'s in cell parameters are only used when they are defined by crystal symmetry. An approximate (isotropic) treatment of cell e.s.d.'s is used for estimating e.s.d.'s involving l.s. planes.
Refinement. Refinement of *F*^2^ against ALL reflections. The weighted *R*-factor *wR* and goodness of fit *S* are based on *F*^2^, conventional *R*-factors *R* are based on *F*, with *F* set to zero for negative *F*^2^. The threshold expression of *F*^2^ > σ(*F*^2^) is used only for calculating *R*-factors(gt) *etc*. and is not relevant to the choice of reflections for refinement. *R*-factors based on *F*^2^ are statistically about twice as large as those based on *F*, and *R*– factors based on ALL data will be even larger.

### Fractional atomic coordinates and isotropic or equivalent isotropic displacement parameters (Å^2^)

**Table:**

	*x*	*y*	*z*	*U*_iso_*/*U*_eq_	
Cl1	0.23185 (8)	0.78305 (9)	0.55574 (6)	0.02213 (16)	
N1	0.8031 (3)	0.6811 (3)	0.5363 (2)	0.0172 (4)	
C1	0.6896 (4)	0.7867 (6)	0.4357 (2)	0.0224 (5)	
C2	0.7510 (5)	0.8922 (5)	0.7083 (3)	0.0256 (6)	
C3	0.7409 (4)	0.6944 (4)	0.6644 (3)	0.0187 (5)	
C4	0.8700 (4)	0.5637 (4)	0.7481 (3)	0.0236 (6)	
C5	0.8089 (4)	0.5381 (4)	0.8763 (2)	0.0202 (5)	
C6	0.8940 (4)	0.6384 (4)	0.9782 (3)	0.0228 (6)	
C7	0.8402 (4)	0.6117 (4)	1.0956 (3)	0.0249 (6)	
C8	0.7005 (4)	0.4842 (4)	1.1138 (3)	0.0262 (6)	
C9	0.6134 (4)	0.3862 (4)	1.0127 (3)	0.0281 (6)	
C10	0.6678 (4)	0.4130 (4)	0.8947 (3)	0.0248 (6)	
H1A	0.718 (5)	0.908 (6)	0.446 (3)	0.039 (10)*	
H1D	0.791 (4)	0.560 (5)	0.509 (3)	0.024 (8)*	
H1B	0.558 (4)	0.754 (4)	0.436 (2)	0.018 (7)*	
H1E	0.925 (4)	0.717 (4)	0.541 (3)	0.020 (8)*	
H1C	0.733 (4)	0.747 (5)	0.356 (3)	0.027 (9)*	
H2A	0.650 (5)	0.967 (5)	0.659 (3)	0.026 (9)*	
H2B	0.880 (5)	0.945 (5)	0.718 (3)	0.030 (10)*	
H2C	0.724 (5)	0.895 (5)	0.791 (3)	0.034 (9)*	
H3A	0.622 (5)	0.651 (5)	0.654 (3)	0.023 (8)*	
H4A	0.863 (4)	0.446 (4)	0.701 (3)	0.017 (7)*	
H4B	0.996 (4)	0.626 (4)	0.757 (3)	0.013 (7)*	
H6	0.980 (4)	0.725 (4)	0.965 (3)	0.017 (7)*	
H7	0.900 (4)	0.680 (4)	1.164 (3)	0.021 (7)*	
H8	0.681 (5)	0.457 (5)	1.193 (3)	0.027 (9)*	
H9	0.521 (4)	0.278 (7)	1.025 (3)	0.030 (7)*	
H10	0.611 (4)	0.337 (4)	0.827 (3)	0.017 (8)*	

### Atomic displacement parameters (Å^2^)

**Table:**

	*U*^11^	*U*^22^	*U*^33^	*U*^12^	*U*^13^	*U*^23^
Cl1	0.0193 (3)	0.0208 (3)	0.0275 (3)	−0.0001 (3)	0.0077 (2)	0.0022 (3)
N1	0.0186 (11)	0.0200 (12)	0.0139 (10)	−0.0016 (9)	0.0064 (8)	0.0003 (9)
C1	0.0263 (12)	0.0248 (12)	0.0166 (11)	−0.0002 (17)	0.0055 (9)	0.0004 (15)
C2	0.0345 (17)	0.0268 (15)	0.0167 (14)	0.0038 (13)	0.0082 (12)	−0.0019 (11)
C3	0.0191 (13)	0.0265 (14)	0.0114 (12)	−0.0008 (11)	0.0052 (10)	0.0006 (11)
C4	0.0268 (14)	0.0282 (16)	0.0172 (13)	0.0038 (12)	0.0076 (11)	0.0011 (11)
C5	0.0193 (12)	0.0226 (13)	0.0192 (13)	0.0032 (10)	0.0049 (10)	0.0024 (10)
C6	0.0222 (13)	0.0260 (14)	0.0205 (13)	−0.0036 (12)	0.0041 (10)	0.0017 (11)
C7	0.0266 (14)	0.0293 (15)	0.0184 (13)	−0.0006 (11)	0.0016 (11)	−0.0043 (11)
C8	0.0280 (14)	0.0352 (16)	0.0172 (13)	0.0045 (12)	0.0094 (11)	0.0061 (11)
C9	0.0267 (15)	0.0294 (16)	0.0292 (15)	−0.0050 (12)	0.0070 (12)	0.0057 (12)
C10	0.0254 (14)	0.0270 (14)	0.0222 (13)	−0.0025 (11)	0.0034 (11)	−0.0017 (11)

### Geometric parameters (Å, °)

**Table:**

N1—C1	1.485 (4)	C4—H4A	1.00 (3)
N1—C3	1.510 (3)	C4—H4B	1.00 (3)
N1—H1D	0.93 (3)	C5—C10	1.388 (4)
N1—H1E	0.90 (3)	C5—C6	1.395 (4)
C1—H1A	0.91 (4)	C6—C7	1.386 (4)
C1—H1B	0.97 (3)	C6—H6	0.90 (3)
C1—H1C	0.99 (3)	C7—C8	1.392 (4)
C2—C3	1.518 (4)	C7—H7	0.95 (3)
C2—H2A	1.00 (3)	C8—C9	1.384 (4)
C2—H2B	0.99 (4)	C8—H8	0.90 (3)
C2—H2C	0.94 (3)	C9—C10	1.393 (4)
C3—C4	1.536 (4)	C9—H9	1.05 (4)
C3—H3A	0.89 (3)	C10—H10	0.96 (3)
C4—C5	1.515 (4)		
			
C1—N1—C3	116.4 (2)	C5—C4—C3	113.4 (2)
C1—N1—H1D	104 (2)	C5—C4—H4A	111.2 (18)
C3—N1—H1D	109 (2)	C3—C4—H4A	104.2 (17)
C1—N1—H1E	108.9 (19)	C5—C4—H4B	109.2 (17)
C3—N1—H1E	108.5 (19)	C3—C4—H4B	103.6 (16)
H1D—N1—H1E	110 (3)	H4A—C4—H4B	115 (2)
N1—C1—H1A	109 (2)	C10—C5—C6	118.7 (2)
N1—C1—H1B	107.8 (17)	C10—C5—C4	120.5 (3)
H1A—C1—H1B	116 (3)	C6—C5—C4	120.8 (2)
N1—C1—H1C	106.5 (18)	C7—C6—C5	120.5 (3)
H1A—C1—H1C	108 (3)	C7—C6—H6	120.9 (19)
H1B—C1—H1C	110 (2)	C5—C6—H6	118.5 (19)
C3—C2—H2A	110 (2)	C6—C7—C8	120.5 (3)
C3—C2—H2B	114 (2)	C6—C7—H7	119.6 (19)
H2A—C2—H2B	116 (3)	C8—C7—H7	119.8 (19)
C3—C2—H2C	108 (2)	C9—C8—C7	119.3 (3)
H2A—C2—H2C	106 (3)	C9—C8—H8	122 (2)
H2B—C2—H2C	101 (3)	C7—C8—H8	119 (2)
N1—C3—C2	109.9 (2)	C8—C9—C10	120.2 (3)
N1—C3—C4	106.2 (2)	C8—C9—H9	120.9 (16)
C2—C3—C4	113.9 (3)	C10—C9—H9	118.3 (17)
N1—C3—H3A	104 (2)	C5—C10—C9	120.8 (3)
C2—C3—H3A	113 (2)	C5—C10—H10	120.6 (17)
C4—C3—H3A	109 (2)	C9—C10—H10	118.4 (18)
			
C1—N1—C3—C2	−60.4 (3)	C4—C5—C6—C7	−178.7 (3)
C1—N1—C3—C4	176.0 (3)	C5—C6—C7—C8	0.1 (4)
N1—C3—C4—C5	−171.5 (2)	C6—C7—C8—C9	−1.1 (4)
C2—C3—C4—C5	67.5 (3)	C7—C8—C9—C10	1.2 (5)
C3—C4—C5—C10	82.9 (3)	C6—C5—C10—C9	−0.8 (4)
C3—C4—C5—C6	−97.6 (3)	C4—C5—C10—C9	178.8 (3)
C10—C5—C6—C7	0.8 (4)	C8—C9—C10—C5	−0.2 (5)

### Hydrogen-bond geometry (Å, °)

**Table:**

*D*—H···*A*	*D*—H	H···*A*	*D*···*A*	*D*—H···*A*
N1—H1D···Cl1^i^	0.93 (4)	2.14 (4)	3.069 (2)	179 (4)
N1—H1E···Cl1^ii^	0.90 (3)	2.22 (3)	3.116 (2)	176 (3)

Symmetry codes: (i) −*x*+1, *y*−1/2, −*z*+1; (ii) *x*+1, *y*, *z*.
